# The Environment and Children’s Health Care in Northwest China

**DOI:** 10.1186/1471-2431-14-82

**Published:** 2014-03-27

**Authors:** Leonardo Trasande, Jingping Niu, Juansheng Li, Xingrong Liu, Benzhong Zhang, Zhilan Li, Guowu Ding, Yingbiao Sun, Meichi Chen, Xiaobin Hu, Lung-Chi Chen, Alan Mendelsohn, Yu Chen, Qingshan Qu

**Affiliations:** 1 Department of Pediatrics, New York University School of Medicine, 227 East 30th Street Rm 109, New York, NY 10016, USA; 2 Department of Environmental Medicine, New York University School of Medicine, New York, USA; 3 Department of Population Health, University School of Medicine, New York, NY, USA; 4 NYU Wagner School of Public Service, New York, NY, USA; 5 NYU Steinhardt School of Culture, Education and Human Development, Department of Nutrition, Food & Public Health, New York, NY, USA; 6 Lanzhou University School of Public Health, Lanzhou, Gansu, China

**Keywords:** Children’s environmental health, Practice, Self-efficacy, Survey, Air pollution, Industrializing world

## Abstract

**Background:**

Industrialization in the northwest provinces of the People’s Republic of China is accelerating rapid increases in early life environmental exposures, yet no publications have assessed health care provider capacity to manage common hazards.

**Methods:**

To assess provider attitudes and beliefs regarding the environment in children’s health, determine self-efficacy in managing concerns, and identify common approaches to managing patients with significant exposures or environmentally-mediated conditions, a two-page survey was administered to pediatricians, child care specialists, and nurses in five provinces (Gansu, Shaanxi, Xinjiang, Qinghai, and Ningxia). Descriptive and multivariable analyses assessed predictors of strong self-efficacy, beliefs or attitudes.

**Results:**

960 surveys were completed with <5% refusal; 695 (72.3%) were valid for statistical analyses. The role of environment in health was rated highly (mean 4.35 on a 1-5 scale). Self-efficacy reported with managing lead, pesticide, air pollution, mercury, mold and polychlorinated biphenyl exposures were generally modest (2.22-2.52 mean). 95.4% reported patients affected with 11.9% reporting seeing >20 affected patients. Only 12.0% reported specific training in environmental history taking, and 12.0% reported owning a text on children’s environmental health. Geographic disparities were most prominent in multivariable analyses, with stronger beliefs in environmental causation yet lower self-efficacy in managing exposures in the northwestern-most province.

**Conclusions:**

Health care providers in Northwest China have strong beliefs regarding the role of environment in children’s health, and frequently identify affected children. Few are trained in environmental history taking or rate self-efficacy highly in managing common hazards. Enhancing provider capacity has promise for improving children’s health in the region.

## Background

Industrialization in the People’s Republic of China (PRC) has produced accelerated economic growth and rapid increases in early life (prenatal, infant and early childhood) exposures to outdoor air pollutants. Coal consumption and production have quadrupled between 1980-2010, increasing mercury emissions, with subsequent concerns about fish and rice contamination with methylmercury and implications for early neurodevelopment [1-3]. Another heavy metal, lead, can also be emitted through lead acid battery production, mining, and smelting. These activities have produced many reported outbreaks of childhood lead poisoning, [4-6] and it has been estimated that one-third of Chinese children may have blood lead ≥10 μg/dL [7].

Current and projected exponential increases in automobile usage in China, coupled with similar growth in industrial activity, are likely to produce continued increases in airborne particulates. This phenomenon is of great concern to children’s health, because given their biologically based vulnerability (increased minute ventilation, rapid alveolar multiplication, and greater alveolar multiplication) [8,9] and the well documented associations of particulate matter exposure with preventable health care utilization for respiratory illnesses [10,11].

Industrialization in China was most intense in the eastern part in the 1980s and 1990s, but since 2000, rapid transformation has ensued especially in the northwest provinces of China as part of a new state policy, China’s Western Development [12,13]. Given this ongoing transformation, a cadre of child health providers who understand children’s unique vulnerability are needed to translate knowledge and inform science-based, effective prevention of chronic childhood disease and disability. While child health provider knowledge and capacity to identify and manage environmental exposures has been studied in industrialized countries [14-18], few publications have assessed provider capacity in a transition or developing world context [19].

We therefore surveyed child health care providers in Northwest China to assess their attitudes and beliefs regarding the role of the environment in children’s health, to determine their self-efficacy in managing environmental health concerns, and to identify commonly used approaches to managing and referring patients with significant exposures or diseases of environmental origin.

## Methods

### Survey instrument

We developed a two-page survey [Additional file 1], modeled on a similar instrument used to assess pediatrician self-efficacy in managing environmental exposures in Michigan, [18] and following the survey methodology outlined by Zonfrillo and Wiebe [20]. Surveys were adapted by coauthors (JN, JL, XL, BZ, ZL, GD, YS, XH, QQ) with substantial clinical and public health experience in China, reworded and reframed for appropriate cultural context, back-translated for accuracy and pilot-tested with practicing health care providers prior to field implementation.

Sixteen questions were divided into three sections. The first asked providers to rate their agreement with a series of belief statements on a Likert scale of 1-5, from “strongly disagree” to “strongly agree”. These questions asked providers to evaluate their perceptions about the role of the environment in children’s health, the need for environmental history taking, and their ability to control environmental exposures. Providers were also asked to opine whether environmentally mediated disease in children was increasing, and whether environmental history taking as part of routine well-child care would take up too much time. The first section also ascertained respondents’ perceived self-efficacy in managing lead, pesticide, air pollution, mercury, mold, and polychlorinated biphenyl (PCB) exposures.

The second section of the survey asked providers to assess whether they had seen a child affected by one or more categories of environmental exposures (e.g., housing, second-hand smoke, pets, air pollution, arsenic, nitrates, mercury) in the past year. For comparison, respondents were also asked to select whether they had seen a child affected by one or more non-environmental concerns (e.g., diet/nutrition, behavior, immunizations) in the past year. A subsequent question asked participants to quantify how many children they had seen in the past year affected by the environmental exposures identified in the previous question, and to quantify how many patients they might refer to a clinic focused on environmental health concerns.

They were asked whether they owned a copy of “Environment and Children Health” published in 2006 by People’s Medical Publishing House, or “Children Environmental Health” published in 2011 by Chongqing University Publishing House, and if so, how often they referred to their book in clinical practice. They were asked whether they had received specific training in environmental history taking, and whether they would be interested in additional training. The final section of the survey asked respondents whether the provider was currently seeing patients, the number of years in practice (not including residency), type of practice (primary care, urgent care, specialty), practice setting (public/community clinic/hospital, private practice, teaching, research, specialty), percent of patient population on low-income family medical insurance or publically-funded assistance, gender, age and zip code.

The survey instrument was translated into Mandarin by native speakers and back-translated to confirm accuracy. This research involving human subjects was performed in accordance with the Declaration of Helsinki, and the survey was approved by the NYU School of Medicine and Lanzhou University School of Public Health IRBs, with a waiver of signed consent.

### Participant identification and recruitment

**Figure 1:**
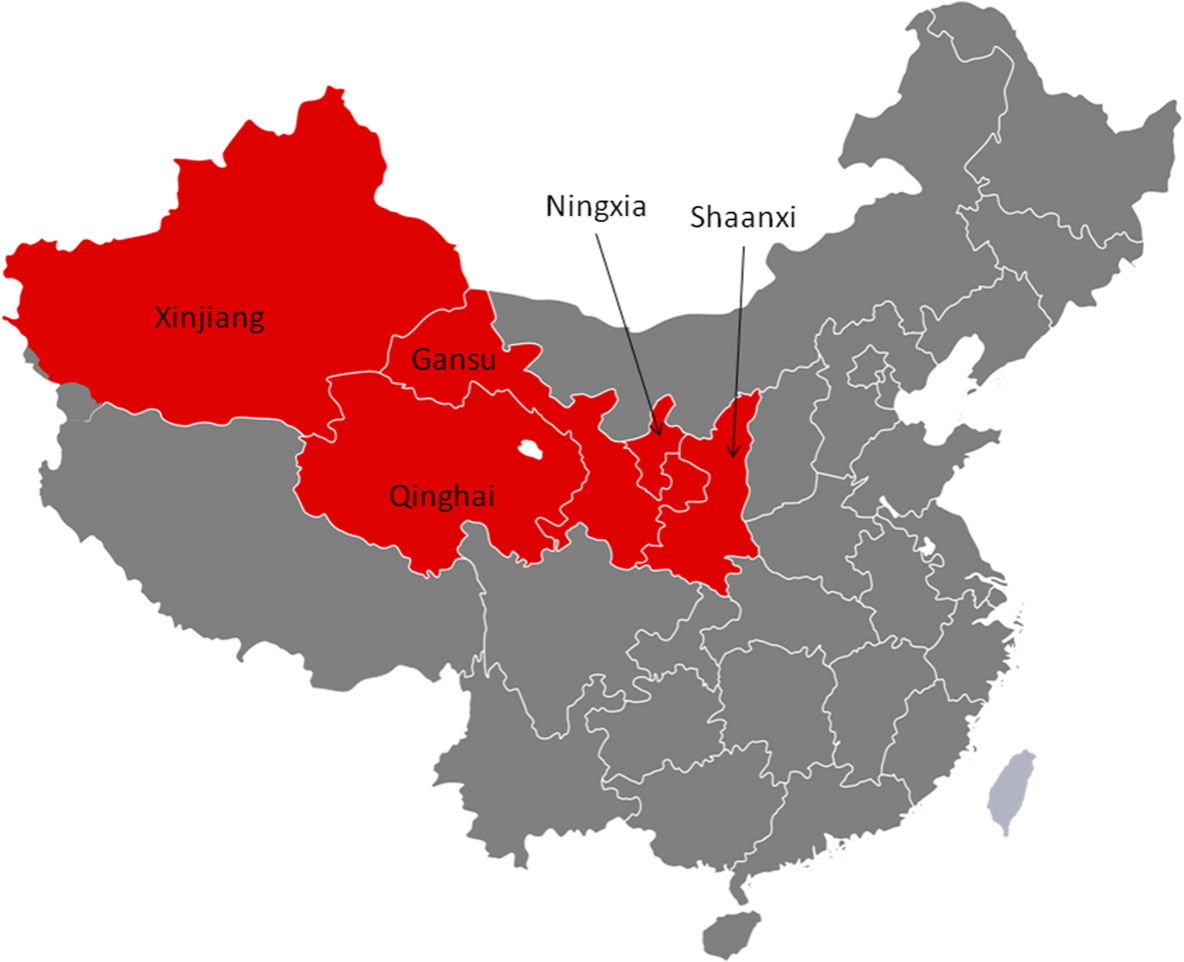
Map of Northwest China (red) with Provinces surveyed.

Our study focused on pediatricians, health professionals who provide preventive services to children, and nurses in five provinces (Gansu, Shaanxi, Xinjiang, Qinghai, and Ningxia; see Figure 1). We identified potential participants through major health care institutions and providers in the region including but not limited to children’s hospitals, provincial maternal and child care institutions, Chinese Medical Association, Chinese Association of Preventive Medicine, Chinese Association of Environmental Science and Chinese Nursing Association. Research assistants and students traveled to the health care providers identified through these networks to request possible participation, and to facilitate completion of the questionnaire.

### Statistical analysis

During data entry, we identified missing values and excluded them from the data analysis. We also checked data by running frequencies to check for outliers and data entry errors, and we randomly sampled and checked 10% of the questionnaires for accuracy. Descriptive data are presented, and multivariable analyses were performed to assess predictors of strong self-efficacy, beliefs or attitudes. For all Likert scales, multinomial logistic analyses were performed to predict odds of higher (or lower) beliefs/attitudes/practices towards the environment and children’s health, in relation to each of the following: age, gender, province, years in practice, practice type, percent public assistance and previous training in environmental history taking (except when previous training was the outcome), while controlling for all other variables. All statistical analyses were conducted using Stata 12.0 (College Station, TX).

### Human subjects protection

This research was reviewed and approved by Institutional Review Boards at NYU School of Medicine and Lanzhou University School of Public Health, and complied with the Helsinki Declaration.

## Results

A total of 960 questionnaires were returned back among pediatricians, child care specialists, and nurses in the Northwest region provinces of Gansu, Shaanxi, Xinjiang, Qinghai, and Ningxia. Refusals across the five provinces were negligible (<5%). We excluded 169 because they were not currently seeing patients, while another 82 were excluded because they were unable to report the number of years that they had been providing health care to children (because they were clinical interns), and 14 did not report age, gender or practice type. As a result, 695 questionnaires (72.3%) were valid for statistical analyses.

Descriptive presentation of our study population is provided in Table 1. The mean age of our study population was 33.6 years, 41% were female, and 36.4% were primary care providers. On average, providers reported 52.7% of patient populations receiving public assistance. Though we endeavored to obtain equal numbers of responses across the five provinces, a substantial number of responses of the incomplete responses were from Xinjiang province (n = 109), leaving representation from that province more modest than the others.

**Table 1:** Description of respondents and their practices

**Characteristic**	**No.**	**Percent**
Age (mean ± SD)	33.6 ± 8.0	
Years in practice (mean ± SD)	7.4 ± 7.1	
Percent public assistance (mean ± SD)	52.7 ± 30.5	
Sex		
Male	410	59.0
Female	285	41.0
Practice type		
Primary care	253	36.4
Urgent care/Emergency	43	6.2
Other (specialty)	399	57.4
Practice setting		
Public/community clinic	669	96.5
Private	5	0.7
Teaching	2	0.3
Research	1	0.1
Other (Specialty)	16	2.3
Province		
Gansu	268	38.6
Shaanxi	149	21.4
Xinjiang	58	8.4
Qinghai	102	14.7
Ningxia	118	17.0
Previous training in environmental history taking	83	12.0

The role of environment in health was reported to be strong (mean 4.35 on a 1-5 Likert scale, Table 2) and environmental history taking was also recognized as very important (mean 3.88). Control that providers had over exposures was rated more modestly (mean 2.79). Self-efficacy reported with managing lead, pesticide, air pollution, mercury, mold and polychlorinated biphenyl exposures were generally modest (2.22-2.52 mean).

**Table 2:** Providers’ self-reported beliefs and self-efficacy regarding environmental health

**Belief statements**	**Mean ± SD**
The role of environmental health impacts on children is of little importance (1) ➔ of great importance (5) (n = 695)	4.35 ± .88
The amount of control child health providers have over environmental health hazards is minimal (1) ➔ maximal (5) (n = 692)	2.79 ± 1.26
The magnitude of children’s environmental related-illnesses is decreasing (1) ➔ increasing (5) (n = 693)	3.89 ± 1.13
Assessing environmental exposures through history-taking in pediatric practice is of little importance (1) ➔ of great importance (5) (n = 695)	3.88 ± 1.07
Conducting an environmental health history on all my patients (1) takes up too much time ➔ does not take up too much time (5) (n = 693)	2.70 ± 1.22
Self efficacy statements	Mean ± SD
How confident are you in managing:	
Lead exposure (n = 689)	2.34 ± 1.26
Pesticide exposure (n = 689)	2.63 ± 1.36
Air pollution exposure (n = 688)	2.22 ± 1.27
Mercury exposure (n = 683)	2.45 ± 1.25
Mold exposure (n = 688)	2.52 ± 1.25
PCB exposure (n = 688)	2.27 ± 1.25

**Figure 2:**
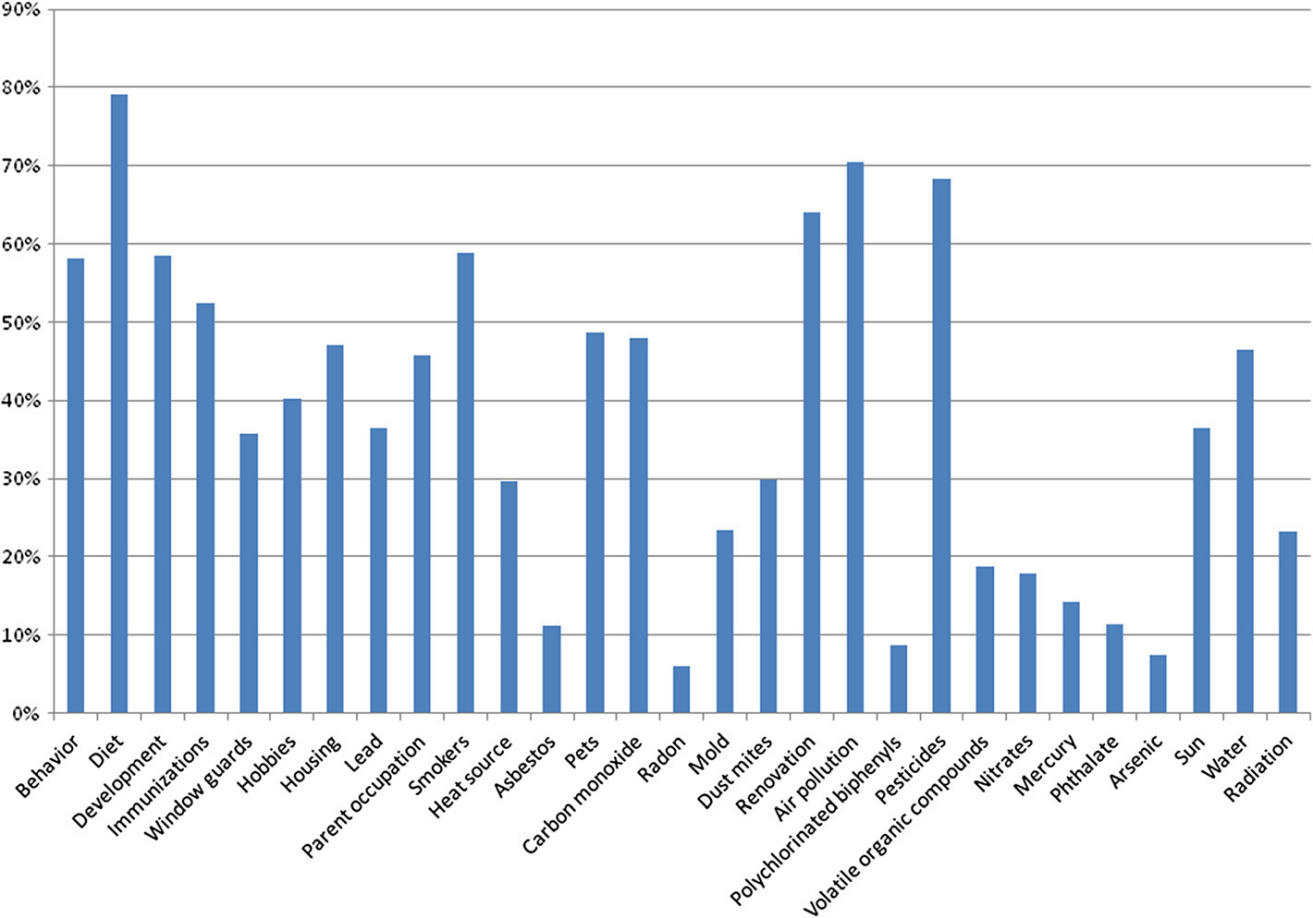
Frequencies of provider report of environmental health and other concerns.

Air pollution (70.5%), pesticides (68.3%) and interior design, renovation and decoration (64.1%) were most frequently identified as major concerns frequently emerging in practices across the five provinces (Figure 2), comparable to general pediatric concerns such as behavior (58.1%), development (58.4%), immunizations (52.4%) and diet (79.1%). Second-hand smoke was more prominent in Qinghai and Ningxia provinces (67.6-71.1%), followed by Gansu and Shaanxi provinces (55.2-57.7%) and Xinjiang province (37.9%). Arsenic was more prominent as a concern in Xinjiang (15.5%) and Shaanxi (14.7%) provinces compared with the others (1.4-9.5%), and water contamination was more prominent in Qinghai (55.8%) and Ningxia (52.5%) provinces than in the other provinces (18.5-38.9%). Air pollution (43%) and lead (24.1%) were less frequent concerns in Xinjiang province than in the others (61.6-86.6% for air pollution and 44.9-50.3% in the others for lead).

95.4% reported having had an experience with patients effected by environmental exposures with 11.9% reporting having seen >20 affected patients in their practice (Table 3). 91.2% would make at least one referral to a specialized clinic for environmental health concerns if it were available. Only 12.0% reported specific training in environmental history taking, and 12.0% reported having a copy of one of two widely-published texts on children’s environmental health.

**Table 3:** Frequencies of child health care provider activities regarding environmental health

**Clinical activities**	**Number (Percent)**
Own environmental health book	83 (12.0)
How many patients were affected in the past year? (n = 692)	
None	32 (4.6)
one patient	32 (4.6)
2-5 patients	312 (45.1)
6-10 patients	135 (19.5)
11-20 patients	99 (14.3)
>20 patients	82 (11.9)
Would refer patients to referral clinic for evaluation and treatment of pediatric environmental health concerns (n = 694)	
Would refer no patients	61 (8.8)
Would refer one patient/year	22 (3.2)
Would refer 2-5 patients/year	212 (30.6)
Would refer 6-10 patients/year	142 (20.5)
Would refer 11-20 patient/year	70 (10.1)
Would refer >20 patients/year	187 (26.7)

Multivariable analyses identified remarkable geographic differences in attitudes towards the environment, especially with respect to the northwestern-most province of Xinjiang. Providers from that province felt more strongly that the role of the environment in children’s health was significant (Table 4), that child health providers had control over environmental hazards, and that assessing the environmental history was important. Interestingly, providers from Xinjiang also were more likely to agree that the environmental history takes too much time. Conversely, providers from Shaanxi felt that taking the environmental history was less important, that the environment does not play as strong a role and that the environmental history does not take too much time. Child health providers from Qinghai also felt that environmental history taking was less important and that the role of environment in health was weaker. More experienced providers also felt more strongly that the environmental history was important, while specialists felt they had less control over environmental hazards than primary care providers. Providers with previous environmental history taking felt stronger control over environmental hazards.

**Table 4:** Significant multivariable predictors of attitudes towards the environment and children’s health

**Outcome**	**Predictor (comparison group)**	**Odds of one point increase in Likert score (95% CI)**
The role of environmental health impacts on children is maximal	Shaanxi providers (compared with Gansu providers)	0.65 (0.44, 0.97)
The role of environmental health impacts on children is maximal	Xinjiang providers (compared with Gansu providers)	18.5 (2.40, 141)
The role of environmental health impacts on children is maximal	Qinghai providers (compared with Gansu providers)	0.49 (0.24, 0.99)
Control child health providers have environmental health hazards is maximal	Xinjiang providers (compared with Gansu providers)	18.6 (6.91, 49.8)
Control child health providers have environmental health hazards is maximal	Specialty providers (compared with primary care providers)	0.49 (0.35, 0.70)
Control child health providers have environmental health hazards is maximal	Training in previous environmental history taking	1.94 (1.17, 3.22)
The magnitude of children’s environmental related-illnesses is increasing	Xinjiang providers (compared with Gansu providers)	11.3 (3.64, 35.0)
Assessing environmental exposures through history-taking in pediatric practice is of great importance	Xinjiang providers (compared with Gansu providers)	5.73 (2.16, 15.2)
Assessing environmental exposures through history-taking in pediatric practice is of great importance	Shaanxi providers (compared with Gansu providers)	0.48 (0.33, 0.71)
Assessing environmental exposures through history-taking in pediatric practice is of great importance	Qinghai providers (compared with Gansu providers)	0.38 (0.19, 0.74)
Assessing environmental exposures through history-taking in pediatric practice is of great importance	Practice years	1.05 (1.01, 1.10)
Conducting an environmental health history on all my patients does not take up too much time	Xinjiang providers (compared with Gansu providers)	0.14 (0.06, 0.32)
Conducting an environmental health history on all my patients does not take up too much time	Shaanxi providers (compared with Gansu providers)	1.47 (1.01, 2.12)

For all Likert scales, multinomial logistic analyses were performed to predict odds of higher (or lower) beliefs/attitudes/practices towards the environment and children’s health, in relation to each of the following: age, gender, province, years in practice, practice type, percent public assistance and previous training in environmental history taking (except when previous training was the outcome), while controlling for all other variables. Results not listed imply p > 0.05.

Providers from Xinjiang also felt less confident in managing lead (Table 5, mercury, pesticide, air pollution, mold and PCB exposures. Ningxia providers also felt less confident in managing lead and air pollution exposures, and Qinghai providers felt less confident managing lead exposures. Providers with previous environmental history taking felt greater efficacy over all exposures (OR 1.99-2.72).

**Table 5:** Significant multivariable predictors of self-efficacy in managing environmental exposures

**Exposure**	**Predictor (comparison group)**	**Odds of one point increase in Likert score (95% CI)**
Lead	Xinjiang providers (compared with Gansu providers)	0.16 (0.06, 0.38)
Lead	Qinghai providers (compared with Gansu providers)	0.36 (0.18, 0.74)
Lead	Ningxia providers (compared with Gansu providers)	0.53 (0.32, 0.89)
Lead	Training in previous environmental history taking	1.99 (1.23, 3.22)
Mercury	Xinjiang providers (compared with Gansu providers)	0.11 (0.05, 0.27)
Mercury	Training in previous environmental history taking	2.12 (1.30, 3.47)
Pesticide	Xinjiang providers (compared with Gansu providers)	0.10 (0.04, 0.26)
Pesticide	Training in previous environmental history taking	2.52 (1.54, 4.11)
Air pollution	Xinjiang providers (compared with Gansu providers)	0.16 (0.06, 0.39)
Air pollution	Ningxia providers (compared with Gansu providers)	0.53 (0.31, 0.92)
Air pollution	Training in previous environmental history taking	2.70 (1.65, 4.44)
Mold	Xinjiang providers (compared with Gansu providers)	0.13 (0.05, 0.33)
Mold	Training in previous environmental history taking	2.23 (1.38, 3.61)
PCB	Xinjiang providers (compared with Gansu providers)	0.17 (0.06, 0.42)
PCB	Training in previous environmental history taking	2.72 (1.67, 4.42)

For all Likert scales, multinomial logistic analyses were performed to predict odds of higher (or lower) beliefs/attitudes/practices towards the environment and children’s health, in relation to each of the following: age, gender, province, years in practice, practice type, percent public assistance and previous training in environmental history taking (except when previous training was the outcome), while controlling for all other variables. Results not listed imply p > 0.05.

Shaanxi (Table 6) and Ningxia providers reported more affected children than providers from other provinces. Xinjiang suggested that they would make fewer referrals than providers from other provinces, while Xinjiang, Shaanxi and Ningxia providers were all more likely to have an environmental health book than providers from Gansu and Qinghai. Providers from Xinjiang were also more likely to have training in environmental history taking, as did specialty providers. Providers with previous environmental history taking were more likely to identify a greater number of affected patients, make hypothetical referrals to an environmental health clinic and own an environmental health book. Providers serving a greater percentage of public patients were less likely to have environmental history training.

**Table 6:** Significant multivariable predictors of behaviors in managing environmental exposures

**Outcome**	**Predictor (comparison group)**	**Odds of one point increase in category (95% CI)**
Number of affected children	Shaanxi providers (compared with Gansu providers)	2.83 (1.91, 4.19)
Number of affected children	Ningxia providers (compared with Gansu providers)	2.59 (1.55, 4.34)
Number of affected children	Training in previous environmental history taking	2.04 (1.24, 3.36)
Number of referrals	Xinjiang providers (compared with Gansu providers)	0.29 (0.14, 0.58)
Number of referrals	Training in previous environmental history taking	2.20 (1.35, 3.59)
Own environmental health book	Shaanxi providers (compared with Gansu providers)	3.42 (1.57, 7.44)
Own environmental health book	Xinjiang providers (compared with Gansu providers)	43.3 (13.2, 142)
Own environmental health book	Specialty providers (compared with primary care providers)	0.17 (0.08, 0.38)
Own environmental health book	Training in previous environmental history taking	2.41 (1.02, 5.67)
Environmental health training	Xinjiang providers (compared with Gansu providers)	2.64 (1.30, 3.81)
Environmental health training	Specialty providers (compared with primary care providers)	2.91 (1.08, 7.81)
Environmental health training	Percent public patients	0.99 (0.97, 0.997)

For all Likert scales, multinomial logistic analyses were performed to predict odds of higher (or lower) beliefs/attitudes/practices towards the environment and children’s health, in relation to each of the following: age, gender, province, years in practice, practice type, percent public assistance and previous training in environmental history taking (except when previous training was the outcome), while controlling for all other variables. Results not listed imply p > 0.05.

## Discussion

This manuscript describes health care providers in Northwest China to have strong beliefs regarding the role of environment in children’s health, frequent identification of children affected by environmental hazards, and gaps in training and self-efficacy in managing many environmental hazards commonly experienced in the region. These findings suggest opportunities to enhance provider capacity to identify harmful and preventable exposures and train health care providers in identifying diseases of environmental origin.

Qualitative comparison with previous surveys suggests similar attitudes and beliefs to those identified in US surveys of pediatricians [14-19], though there is notably lower self-efficacy for managing lead exposures, which is of great concern if indeed prevalence of elevated blood lead levels is in the range of 30%, as previously suggested [7]. Self-efficacy for other exposures was not qualitatively different, nor were attitudes towards children’s environmental health or frequency of training in environmental history taking.

Survey response rates were high, though incomplete surveys were more frequent in Xinjiang, and so the usual caveats about selection bias and external validity to the population of child health providers apply. Though there was a waiver of informed consent, concerns about identifiability with respect to their attitudes may have limited respondent candidness, and there may have been a tendency to give socially appropriate answers. Provider self-efficacy does not necessarily translate into appropriate care, and volumes of affected patients and hypothetical referrals may be underestimates due to the modest self-efficacy identified for many exposures.

Data are not available on the number of providers in Northwest China, and our use of professional societies and institutions to identify potential participants may have skewed our results towards providers with stronger understanding of emerging issues in environmental health. Assessing validity of self-assessed efficacy is also very difficult, as even basic assessments of children’s environmental health proficiency have not yet been developed. Further research is needed in developing such assessment tools.

The geographic diversity in self-efficacy and attitudes is striking. Though the stronger attitudes could be explained by selection bias towards those most interested and trained in environmental health, the lower self-efficacy in those same regions despite controlling for provider training cannot. We also identified an interesting discrepancy in that providers from that region held stronger beliefs in causation of environmental hazards, and were more likely to be trained in environmental history taking, yet were more likely to state that the environmental history took too much time. This could be interpreted to suggest that stronger beliefs in the role of environment in health led to greater inquiry into these concerns, competing with other concerns in busy clinical and public health practices. Of note, providers from the most northwest province did not report a greater volume of patients affected by environmental exposures.

Weaker attitudes towards the role of the environment in health in Shaanxi and Qinghai provinces raise additional concerns, because these same providers voiced weaker self-efficacy in management of lead hazards. Few owned a book on environmental health or had training in environmental history taking, and self-efficacy was low for all hazards queried. Yet, there is some hope in that providers with training consistently voiced stronger self-efficacy in managing hazards and more frequently reported identifying affected children.

The differences may also represent diversity in exposures across these five provinces which span a huge geographic region, bounded on three sides by Kazakhstan, Kyrgyzstan, Tajikstan, Afghanistan, Pakistan, India, Tibet and Mongolia. Gansu is known for being home to the world’s second largest nickel refinery [21], while Shaanxi has one of the most rapidly growing urban centers in China (Xi’an). Qinghai is home to iron, steel and oil industries [22], while Ningxia is known for medicinal, chemical and wine production [23].

The findings in this manuscript will form the basis for an educational conference, which will allow us to explore better needs identified in the survey, as well as gaps and barriers to effective application of scientific knowledge to drive policy to protect children from air pollution hazards. Child health providers, community stakeholders and decision makers will be invited to attend, and they will be encouraged to ask others to join. The focus of the conference will be on outdoor air pollution, and additional sessions will provide context for other environmental exposures to which children are vulnerable. Surveys at the initiation of the conference will be used to quantify pre-conference knowledge and attitudes towards children and environmental factors (especially air pollution) and will be followed by post-test surveys to determine knowledge gained from the conference.

## Conclusions

Health care providers in Northwest China have strong beliefs regarding the role of environment in children’s health, and frequently identify children affected by environmental hazards. Few are trained in environmental history taking or rate their self-efficacy highly in managing many environmental hazards commonly experienced in the region. Enhancing provider capacity to identify harmful and preventable exposures has promise for improving children’s health in the region.

## Abbreviations

OR: Odds ratio; PRC: People’s Republic of China.

## Competing interests

The authors declare that they have no competing interests.

## Authors’ contributions

LT and JN designed the study, wrote initial drafts of the manuscript, obtained funding and submitted human subjects approvals. QQ, JL, XL, BZ, ZL, GD designed, translated and pilot tested surveys. YS, MX and XH oversaw recruitment and survey administration. L-C C, AM, YC, GD and ZL participated in data analyses and reviewed manuscript drafts. All authors read and approved the final manuscript.

## Pre-publication history

The pre-publication history for this paper can be accessed here:

http://www.biomedcentral.com/1471-2431/14/82/prepub

## Supplementary Material

Additional file 1 Survey of Child Health Providers. Click here for file
