# Supplementary material for: The Environment and Children’s Health Care in Northwest China
Source: BMC Pediatr. 2014 Mar 27;14:82. doi: 10.1186/1471-2431-14-82 (PMC3986873; doi:10.1186/1471-2431-14-82)
Supplement: Additional file 1 — Survey of Child Health Providers. [file 1471-2431-14-82-S1.docx]

Environmental Health Concerns in Pediatric Practice

*Please notice the survey is printed on the front and back of the paper.*

*This is a three-part survey and will take five to seven minutes to complete.*

For the purpose of this survey, environmental health refers to those aspects of human health and disease that are determined by factors in the environment, and to assessing and controlling factors in the environment that can affect health.

1. Please circle one answer for the following questions.

|  |  |  |  |  |  |
| --- | --- | --- | --- | --- | --- |
| The role of environmental health impacts on children is | of little importance  1 | 2 | 3 | 4 | of great importance  5 |
| The control pediatricians have over environmental health hazards is | minimal  1 | 2 | 3 | 4 | maximal  5 |
| The magnitude of children’s environmental related-illnesses is | decreasing  1 | 2 | 3 | 4 | increasing  5 |
| Assessing environmental exposures through history-taking in pediatric practice is | of little importance  1 | 2 | 3 | 4 | of great importance  5 |
| Conducting an environmental health history as part of routine well-child care | Takes too much time  1 | 2 | 3 | 4 | Does not take too much time  5 |

2. Please circle how confident you are in dealing with the exposures below.

|  | Not confident |  |  |  | Very confident |
| --- | --- | --- | --- | --- | --- |
| Lead exposure  Pesticide exposure  Air pollution exposure  Mercury exposure  Mold exposure  PCB (polychlorinated biphenyl) exposure | 1  1  1  1  1  1 | 2  2  2  2  2  2 | 3  3  3  3  3  3 | 4  4  4  4  4  4 | 5  5  5  5  5  5 |

3. Please check off whether you have seen children affected by any of the following in the past year:

| General Anticipatory Guidance | Home Environment | Outdoor Environment |
| --- | --- | --- |
| - Behavior - Diet/nutrition - Development - Firearms in the home - Immunizations - Window guards/injury prevention - Hobbies of parents and child | - Housing (age, type) - Lead - Parents’/teens’ occupations - Smokers around the child - Heat source in the home - Asbestos - Pets - Carbon Monoxide - Radon - Mold - Dust mites, cockroaches | - Air pollution - Polychlorinated biphenyls (PCBs) - Pesticides - Volatile organic compounds (VOCs) - Nitrates - Mercury - Phthalate plasticizers - Arsenic (including in playground equipment) - Sun exposure - Water quality (including source of water) - Radiation exposure (including Potassium Iodide) |

4. How many patients have been affected by the environmental exposures listed in question 3?

- None
- One
- Two to Five
- Six to Ten
- Ten to Twenty
- Twenty or More

5. If there were a clinic where you could refer patients for clinical evaluation and treatment of their environmental health concerns, how many of the patients would you refer to the clinic per year?

- None
- One
- Two to Five
- Six to Ten
- Ten to Twenty
- Twenty or More

6. Do you have a copy of “Environment and Children Health” published in 2006 by People’s Medical Publishing House, or “Children Environmental Health” published in 2011 by Chongqing University Publishing House?

- Yes
- No

7. If so, how often do you refer to either of these books?

- Daily
- Weekly
- Once a month
- Once every few months
- Yearly
- Never

8. Have you received any specific training in environmental history-taking?

□ Yes □ No

9. Are you currently seeing patients?

□ Yes □ No

10. How many years have you been practicing in pediatrics (not including residency)?

__________years

11. What is your type of practice?

□ Primary care □ Urgent care/Emergency □ Specialty ______________________________

(please specify)

12. What best describes your practice setting the majority of the time?

□ Public or community clinic, hospital, or health center □ Private practice

□ Teaching □ Research □ Specialty ______________________________

(please specify)

13. What percentage of your patient population is on Low Income family medical insurance or public-funded assistance?

_______%

14. What is your gender?

□ Female □ Male

15. What is your age?

__________

16. Please provide your zip code:

___ ___ ___ ___ ___ ___
